# Supplementary material for: Loss of CXC-Chemokine Receptor 1 Expression in Chorioamnionitis Is Associated with Adverse Perinatal Outcomes
Source: Diagnostics (Basel). 2022 Apr 1;12(4):882. doi: 10.3390/diagnostics12040882 (PMC9028796; doi:10.3390/diagnostics12040882)
Supplement: Supplementary file 1 [file diagnostics-12-00882-s001.zip › diagnostics-1613305-supplementary.pdf]

Supplementary Table S1. List of 101 cases of chorioamnionitis with or without funisitis showing the intensity of CXCR1 immunoexpression in AEC, DC, UCEC and UCSMW, and the perinatal outcomes

	Maternal age	Ethnicity	Gestational age	MIR	FIR	AEC	DC	UCEC	UCSMW	Perinatal outcomes
1	28	Malay	16	Stage 2	Stage 0	1+	NA	1+	2+	Miscarriage
2	26	Chinese	39	Stage 2	Stage 0	1+	2+	1+	3+	None
3	27	Malay	36	Stage 2	Stage 0	1+	2+	2+	2+	None
4	31	Malay	39	Stage 2	Stage 2	1+	NA	1+	2+	None
5	27	Malay	39	Stage 2	Stage 2	-	3+	1+	3+	None
6	24	Malay	37	Stage 2	Stage 2	0	3+	1+	2+	None
7	30	Malay	39	Stage 1	Stage 1	2+	NA	1+	2+	None
8	37	Malay	38	Stage 2	Stage 0	1+	3+	1+	2+	None
9	35	Malay	33	Stage 2	Stage 2	0	3+	1+	3+	RDS
10	33	Chinese	39	Stage 2	Stage 2	2+	3+	2+	2+	None
11	32	Malay	38	Stage 2	Stage 1	1+	1+	2+	2+	None
12	31	Malay	32	Stage 2	Stage 2	2+	3+	2+	2+	None
13	29	Malay	39	Stage 2	Stage 0	2+	3+	2+	2+	None
14	27	Malay	22	Stage 2	Stage 0	1+	2+	0	3+	Miscarriage
15	24	Malay	38	Stage 2	Stage 1	-	3+	1+	2+	None
16	30	Malay	37	Stage 2	Stage 1	1+	2+	1+	2+	None
17	29	Chinese	38	Stage 2	Stage 1	1+	1+	1+	1+	None
18	27	Malay	38	Stage 1	Stage 0	1+	NA	1+	2+	None
19	37	Chinese	38	Stage 1	Stage 1	0	3+	1+	2+	None
20	25	Indian	39	Stage 2	Stage 2	2+	NA	2+	3+	None
21	33	Malay	35	Stage 2	Stage 0	1+	2+	2+	2+	None
22	34	Chinese	39	Stage 2	Stage 0	1+	NA	1+	2+	None
23	29	Malay	38	Stage 1	Stage 2	1+	NA	1+	2+	None
24	23	Chinese	39	Stage 1	Stage 2	1+	NA	0	1+	None
25	30	Malay	38	Stage 1	Stage 0	0	NA	1+	2+	None
26	32	Chinese	38	Stage 2	Stage 2	2+	3+	1+	2+	Neonatal jaundice
27	39	Malay	38	Stage 1	Stage 2	1+	NA	1+	0	None
28	26	Malay	40	Stage 2	Stage 2	0	NA	3+	3+	None
29	28	Malay	40	Stage 2	Stage 2	0	2+	1+	1+	None
30	30	Others	38	Stage 2	Stage 2	1+	2+	1+	2+	None
31	33	Malay	39	Stage 2	Stage 1	2+	NA	2+	2+	None
32	26	Malay	38	Stage 1	Stage 0	2+	3+	2+	3+	Pneumonia
33	40	Malay	39	Stage 1	Stage 1	-	2+	0	1+	None
34	26	Malay	38	Stage 1	Stage 1	2+	2+	2+	2+	None
35	28	Malay	38	Stage 3	Stage 2	-	3+	2+	2+	Meconium aspiration syndrome
36	33	Malay	39	Stage 2	Stage 0	1+	NA	1+	2+	None
37	25	Malay	38	Stage 2	Stage 2	1+	3+	1+	2+	None
38	27	Malay	38	Stage 2	Stage 0	0	0	0	2+	None

39	36	Malay	37	Stage 2	Stage 2	1+	1+	0	2+	None
40	27	Malay	38	Stage 2	Stage 0	1+	3+	2+	2+	None
41	32	Chinese	39	Stage 2	Stage 1	1+	2+	0	1+	None
42	27	Chinese	39	Stage 2	Stage 1	1+	NA	1+	1+	None
43	30	Malay	31	Stage 2	Stage 2	0	3+	0	1+	Neonatal death
44	37	Chinese	39	Stage 2	Stage 3	0	NA	1+	2+	None
45	41	Chinese	39	Stage 2	Stage 0	-	3+	2+	2+	None
46	29	Malay	39	Stage 3	Stage 0	0	0	0	2+	IUD
47	25	Chinese	40	Stage 2	Stage 1	0	2+	1+	2+	None
48	31	Others	38	Stage 2	Stage 0	2+	3+	1+	2+	None
49	35	Malay	40	Stage 2	Stage 2	1+	3+	1+	2+	None
50	26	Chinese	38	Stage 2	Stage 1	1+	3+	2+	3+	None
51	34	Malay	39	Stage 3	Stage 2	-	2+	1+	2+	None
52	33	Chinese	38	Stage 2	Stage 1	1+	NA	1+	2+	None
53	32	Malay	37	Stage 2	Stage 1	0	2+	1+	2+	None
54	27	Malay	40	Stage 2	Stage 1	1+	3+	2+	3+	None
55	28	Malay	38	Stage 2	Stage 1	1+	3+	2+	2+	None
56	28	Malay	38	Stage 3	Stage 3	1+	3+	2+	3+	None
57	30	Chinese	38	Stage 1	Stage 1	1+	1+	0	2+	None
58	30	Malay	19	Stage 3	Stage 3	2+	2+	1+	3+	Miscarriage
59	36	Malay	38	Stage 2	Stage 2	2+	3+	1+	2+	Pneumonia
60	27	Malay	38	Stage 2	Stage 1	-	1+	1+	2+	None
61	33	Malay	36	Stage 2	Stage 1	2+	3+	1+	1+	None
62	31	Malay	39	Stage 2	Stage 1	2+	3+	1+	2+	None
63	27	Malay	39	Stage 3	Stage 3	1+	3+	1+	1+	None
64	35	Malay	38	Stage 2	Stage 2	1+	3+	2+	2+	Pneumonia
65	34	Malay	38	Stage 2	Stage 0	2+	2+	2+	3+	None
66	32	Malay	39	Stage 2	Stage 2	2+	NA	2+	3+	None
67	34	Chinese	39	Stage 2	Stage 1	1+	NA	0	0	None
68	28	Malay	38	Stage 1	Stage 0	-	2+	1+	2+	None
69	26	Malay	39	Stage 2	Stage 1	-	1+	1+	1+	None
70	37	Malay	38	Stage 2	Stage 1	1+	1+	0	2+	None
71	28	Malay	38	Stage 2	Stage 1	-	1+	1+	1+	None
72	31	Malay	38	Stage 2	Stage 1	1+	3+	2+	2+	None
73	31	Malay	35	Stage 3	Stage 3	2+	3+	1+	1+	None
74	30	Malay	38	Stage 2	Stage 1	1+	NA	1+	1+	None
75	30	Indian	39	Stage 2	Stage 1	1+	3+	2+	2+	None
76	28	Malay		Stage 2	Stage 1	2+	3+	2+	2+	None
77	28	Chinese	39	Stage 1	Stage 1	-	1+	1+	2+	None
78	28	Malay	34	Stage 2	Stage 0	1+	3+	1+	1+	None
79	26	Malay	32	Stage 1	Stage 0	3+	3+	2+	2+	None
80	28	Malay	38	Stage 1	Stage 0	0	1+	0	2+	None
81	28	Malay	37	Stage 2	Stage 1	1+	3+	2+	2+	None
82	33	Malay	38	Stage 2	Stage 1	0	2+	0	1+	None
83	28	Malay	35	Stage 2	Stage 1	1+	1+	1+	2+	None

84	27	Malay	39	Stage 2	Stage 1	0	1+	0	1+	None
85	27	Malay	40	Stage 3	Stage 2	-	3+	2+	2+	None
86	31	Malay	40	Stage 2	Stage 1	-	1+	1+	2+	None
87	28	Malay	39	Stage 1	Stage 0	1+	1+	0	1+	None
88	26	Others	39	Stage 2	Stage 1	1+	3+	0	2+	None
89	32	Malay	38	Stage 1	Stage 0	1+	3+	1+	1+	None
90	35	Malay	39	Stage 2	Stage 1	-	3+	1+	2+	None
91	30	Malay	36	Stage 1	Stage 0	1+	2+	2+	2+	None
92	30	Chinese	39	Stage 1	Stage 0	1+	3+	0	1+	None
93	39	Malay	38	Stage 1	Stage 0	2+	3+	2+	2+	None
94	33	Malay	38	Stage 2	Stage 2	-	3+	1+	1+	None
95	28	Malay	38	Stage 2	Stage 1	2+	3+	2+	2+	None
96	36	Malay	38	Stage 1	Stage 1	1+	NA	0	1+	None
97		Malay	33	Stage 1	Stage 0	2+	2+	1+	1+	Transient tachypnea of neonate
98	21	Malay	23	Stage 2	Stage 1	1+	1+	0	1+	Miscarriage
99	37	Malay	38	Stage 3	Stage 3	-	2+	1+	2+	None
100	35	Chinese	38	Stage 3	Stage 3	-	2+	2+	2+	None
101	35	Chinese	39	Stage 2	Stage 2	1+	3+	1+	2+	Pneumonia

AECs – Amnion epithelial cells, CXCR1 - CXC-chemokine receptor 1, DCs – Decidual cells, F/N – fetal/neonatal, FIR – Fetal inflammatory response, IUD – Intrauterine death, MIR – Maternal inflammatory response, NA – Not applicable/Not available, Pneum – Pneumonia, RDS – respiratory distress syndrome, UCECs – Umbilical cord endothelial cells, UCBVs – Umbilical cord blood vessel smooth muscle wall

Supplementary Table S2. List of 32 cases of control without chorioamnionitis showing the expression of CXCR1 in AEC, DC, UCEC and UCSMW

No	Age	Ethnicity	Gestational age	MIR	FIR	CXCR1 expression			
						AEC	DC	UCEC	UCSMW
1	36	Malay	37	Stage 0	Stage 0	1+	3+	1+	2+
2	34	Malay	39	Stage 0	Stage 0	3+	3+	3+	3+
3	29	Malay	38	Stage 0	Stage 0	1+	2+	1+	1+
4	34	Malay	38	Stage 0	Stage 0	2+	3+	3+	3+
5	31	Malay	38	Stage 0	Stage 0	1+	3+	2+	2+
6	32	Malay	34	Stage 0	Stage 0	2+	NA	2+	2+
7	35	Malay	39	Stage 0	Stage 0	1+	1+	2+	2+
8	36	Malay	38	Stage 0	Stage 0	1+	2+	1+	2+
9	26	Malay	37	Stage 0	Stage 0	2+	2+	2+	2+
10	44	Chinese	39	Stage 0	Stage 0	1+	NA	1+	3+
11	26	Malay	38	Stage 0	Stage 0	2+	NA	2+	2+
12	34	Malay	36	Stage 0	Stage 0	1+	1+	0	2+
13	24	Malay	39	Stage 0	Stage 0	1+	2+	NA	NA
14	35	Malay	38	Stage 0	Stage 0	1+	2+	NA	NA
15	32	Malay	39	Stage 0	Stage 0	1+	NA	1+	1+
16	32	Malay	37	Stage 0	Stage 0	1+	NA	2+	0

17	24	Iban	28	Stage 0	Stage 0	NA	NA	2+	1+
18	31	Malay	40	Stage 0	Stage 0	0	0	0	0
19	36	Malay	38	Stage 0	Stage 0	1+	1+	NA	NA
20	26	Malay	39	Stage 0	Stage 0	1+	2+	NA	NA
21	37	Malay	35	Stage 0	Stage 0	2+	2+	NA	NA
22	36	Malay	37	Stage 0	Stage 0	0	1+	1+	0
23	33	Malay	39	Stage 0	Stage 0	NA	NA	1+	1+
24	32	Indian	32	Stage 0	Stage 0	2+	2+	NA	NA
25	41	Malay	35	Stage 0	Stage 0	2+	2+	2+	2+
26	29	Malay	39	Stage 0	Stage 0	1+	2+	NA	NA
27	32	Malay	38	Stage 0	Stage 0	2+	NA	1+	2+
28	31	Chinese	38	Stage 0	Stage 0	1+	1+	NA	NA
29	35	Chinese	39	Stage 0	Stage 0	0	0	2+	1+
30	28	Malay	34	Stage 0	Stage 0	0	2+	0	1+
31	35	Malay	35	Stage 0	Stage 0	1+	3+	1+	1+
32	34	Malay	39	Stage 0	Stage 0	1+	3+	2+	2+

AECs – Amnion epithelial cells, FIR – Fetal inflammatory response, MIR – Maternal inflammatory response, NA – Not applicable/Not available, UC ECs – Umbilical cord endothelial cells, UC BV SMW – Umbilical cord blood vessel smooth muscle wall
